# Computational Study on Temperature Driven Structure–Function Relationship of Polysaccharide Producing Bacterial Glycosyl Transferase Enzyme

**DOI:** 10.3390/polym13111771

**Published:** 2021-05-28

**Authors:** Patricio González-Faune, Ignacio Sánchez-Arévalo, Shrabana Sarkar, Krishnendu Majhi, Rajib Bandopadhyay, Gustavo Cabrera-Barjas, Aleydis Gómez, Aparna Banerjee

**Affiliations:** 1 Escuela de Ingeniería en Biotecnología, Facultad de Ciencias Agrarias y Forestales, Universidad Católica del Maule, Talca 3466706, Chile; patricio.gonzalez@alu.ucm.cl (P.G.-F.); ignacio.sanchez@alu.ucm.cl (I.S.-A.); 2 UGC Center of Advanced Study, Department of Botany, The University of Burdwan, Bardhaman 713104, India; runka.sarkar@gmail.com (S.S.); kmkrishnendu@gmail.com (K.M.); rajibindia@gmail.com (R.B.); 3 Unidad de Desarrollo Tecnológico (UDT), Universidad de Concepción, Av. Cordillera 2634, Parque Industrial Coronel, Coronel 3349001, Chile; g.cabrera@udt.cl; 4 Centro de Biotecnología de los Recursos Naturales (CENBio), Facultad de Ciencias Agrarias y Forestales, Universidad Católica del Maule, Talca 3466706, Chile; aleydisgomez1965@gmail.com; 5 Centro de Investigación de Estudios Avanzados del Maule, Vicerrectoría de Investigación y Posgrado, Universidad Católica del Maule, Talca 3466706, Chile

**Keywords:** bacterial polysaccharides, glycosyl transferase, mesophiles, thermophiles, hyperthermophiles, structure-function study

## Abstract

Glycosyltransferase (GTs) is a wide class of enzymes that transfer sugar moiety, playing a key role in the synthesis of bacterial exopolysaccharide (EPS) biopolymer. In recent years, increased demand for bacterial EPSs has been observed in pharmaceutical, food, and other industries. The application of the EPSs largely depends upon their thermal stability, as any industrial application is mainly reliant on slow thermal degradation. Keeping this in context, EPS producing GT enzymes from three different bacterial sources based on growth temperature (mesophile, thermophile, and hyperthermophile) are considered for in silico analysis of the structural–functional relationship. From the present study, it was observed that the structural integrity of GT increases significantly from mesophile to thermophile to hyperthermophile. In contrast, the structural plasticity runs in an opposite direction towards mesophile. This interesting temperature-dependent structural property has directed the GT–UDP-glucose interactions in a way that thermophile has finally demonstrated better binding affinity (−5.57 to −10.70) with an increased number of hydrogen bonds (355) and stabilizing amino acids (Phe, Ala, Glu, Tyr, and Ser). The results from this study may direct utilization of thermophile-origin GT as best for industrial-level bacterial polysaccharide production.

## 1. Introduction

High molecular weight polysaccharides are central constituents of the cell wall and extracellular matrix for all domains of life [1]. Specifically, in bacteria, they are the integrative part of the cell membranes (lipopolysaccharide/LPS), capsule (capsular polysaccharide/CPS), and biofilm (exopolysaccharide/EPS) network. The production of biofilm is among one of the most pertinent defense mechanisms of the bacteria in a potentially extreme environment [1,2]. EPS are carbohydrate polymers that assist the bacterial communities to endure extreme temperature, salinity, nutrient scarcity, or presence of toxic compounds [3]. In recent years, the increased demand for biopolymers for pharmaceutical, food, and other industrial applications has led to a remarkable research interest in polysaccharide biology [4,5,6]. Substantial attention in this regard goes to the isolation and identification of new bacterial polysaccharides that might have innovative applications as gelling, emulsifying, or stabilizing agents [7]. The application of the bacterial EPSs largely depends upon its thermal stability, as any industrial application is largely dependent on slow thermal degradation [8]. Particularly in the light of advanced food industry-related applications, multifunctionality, and thermoplasticity of the bacterial EPSs are two of the most considered factors [9].

A class of enzyme named glycosyltransferase (GTs) is reported to make its expression through GT operon and is responsible for EPS production in bacteria [10]. Actually, it is a wide class of enzymes that catalyze the formation of glycosidic linkage by transferring a sugar residue from a donor substrate to an acceptor. The acceptor substrates are mono-, di-, or oligosaccharides, proteins, lipids, DNA, and numerous other small molecules [11]. The donor substrates are majorly nucleotide-sugar conjugates (~65%) along with some lipid phosphate sugars and phosphate sugars [12]. Therefore, they play key roles in the biosynthesis of oligo and polysaccharides, protein glycosylation, and the synthesis of valuable natural products [11]. According to the carbohydrate-active enzymes classification (CAZy), the GTs (EC 2.4) are currently composed of 97 families [13]. The classification of GT is complex because many families are not reported with any characterized enzymes yet, but this highly diverse class of enzymes is reported to have many opportunities in chemo-enzymatic tailoring of novel natural polymers [12,14]. This precedes the importance of further research related to GTs and EPS production.

Considering the vast applicability and the significance of thermal stability of the bacterial EPSs, it will be particularly interesting to observe the in silico structure–function relationship of the EPS-producing GTs. Another aspect to consider in this particular topic is the temperature-dependent enzymatic stability, as the thermal stability of any enzyme is always crucial in an industrial process [15]. Thermostable and mesostable enzymes have been compared long back, and features associated with improved thermostability are described too. Some characteristics of thermophilic enzymes, such as improved packing consistency [16], improved electrostatic interactions [17], and increased hydrophobicity in the protein center [18], contribute to the stability of protein folding. Other characteristics, such as reduced conformational flexibility or unfolding entropy, reduce destabilizing forces [19]. All the above features together or separately may improve a protein’s thermostability. In this sense, to the best of our knowledge, no previous analyses are there on EPS biopolymer producing GT enzymes from a broad range of temperature-adapted bacterial strains.

Thus, in the present study, the structure–function relationship of the bacterial EPS producing GTs has been unfolded through the help of computational tools. For this, mesophilic, thermophilic, and hyperthermophilic bacteria origin GTs are considered. Though high temperature-origin bacterial bioproducts are already reported for enhanced stability, but in this study, increasing growth temperature ranges (i.e., mesophile, thermophile, and hyperthermophile) are considered for an all-inclusive outlook on protein stability and structural plasticity. A computational study on structure–function relationship determination of GT is novel in its own way, because many GTs considered for prospective carbohydrate catalysis are yet not fully understood, including those of biofilm or EPS formation [1]. Thus, this computational analysis may create a broad horizon to look into the bacterial GT sequences for likely stability, protein–protein interactions, enzyme-substrate affinity, and stability of the EPS produced by them. Hence, overall structural constancy, functional aspects, protein–protein interactions, and enzyme-substrate affinity of the EPS producing GTs have been studied.

## 2. Materials and Methods

### 2.1. Accession of Target Proteins

Target protein sequences of biofilm-producing GT enzymes respectively from mesophilic, thermophilic, and hyperthermophilic bacteria were retrieved from NCBI (National Centre for Biotechnology Information) Protein in both PDB and FASTA format (https://www.ncbi.nlm.nih.gov/protein/?term=, accessed on 1 May 2021). The Protein database (NCBI Protein) has the collection of sequences from various sources, including translations from annotated coding regions in GenBank, RefSeq, and TPA, as well as records from SwissProt, PIR, PRF, and PDB.

### 2.2. Primary Analysis of Physicochemical Parameters and Structure

To obtain the physicochemical parameters of the retrieved proteins, a web-based server, ExPASy-ProtParam (http://web.expasy.org/protparam/, accessed on 1 May 2021) was run. Physicochemical parameters of a protein give an idea on its basic nature. Different physicochemical characters like the number of amino acids contributing to the structure, molecular weight, isoelectric point (pI), grand average of hydropathicity (GRAVY), instability index (II), and aliphatic index (AI) were computed. For getting the detailed primary structure of the query proteins, another computational tool GPMAW (http://www.alphalyse.com/customer-support/gpmaw-lite-bioinformatics-tool/buy-gpmaw/, accessed on 1 May 2021) was used. This tool was employed to predict the nature of common contributing amino acids in the formation of the protein’s structure. Getting the overview of a protein’s primary structure is important as it drives the intramolecular bonding of amino acid chains [20], which ultimately determines the final three-dimensional folding of a protein.

### 2.3. Secondary Structure Assessment

Web-based server SOPMA (Self-Optimized Prediction Method with Alignment) (https://npsa-prabi.ibcp.fr/cgi-bin/npsa_automat.pl?page=npsa_sopma.html, accessed on 1 May 2021) was employed to run the sequences of interest and predict their secondary structure in terms of α-helix, β-turn, extended strand, and random coils. The secondary structure of any protein helps to project the local interaction between amino acid stretches by determining the hydrogen bonds, as hydrogen bonds help in protein folding and achieving structural stability by intermolecular interactions [21]. UCSF Chimera 1.8.1 with a command-line option was used to analyze the total number of hydrogen bonds present in each of the retrieved protein sequences [21].

### 2.4. Assessment of the Three Dimensional Structure, Modeling, and Validation

Three-dimensional structures of the chosen proteins of interest (representing each class of mesophiles, thermophiles, and hyperthermophiles) have been pre-optimized by means of different computational tools before heading to the docking analyses. In order to observe the electrostatic attractions between the positively and negatively charged amino acid residues, salt bridges/ion bridges were evaluated using the ESBRI server (http://bioinformatica.isa.cnr.it/ESBRI/input.html, accessed on 1 May 2021). Another web-based server, ERRAT (http://services.mbi.ucla.edu/ERRAT/, accessed on 1 May 2021) was availed to evaluate the statistics of non-bonded salt bridge interactions among different atoms of the protein sequences [22]. Use of the SAVES server (http://servicesn.mbi.ucla.edu/SAVES/, accessed on 1 May 2021) resulted in the achievement of high-resolution 3D structures of the proteins. Homology 3D modeling of the target sequences has been realized through SWISS-MODEL QMEAN that is based on qualitative model energy analysis (https://swissmodel.expasy.org/qmean/, accessed on 1 May 2021). Based on different scoring approaches available in the workspace, this server estimates the most suitable and best-matched protein structures depending on the model quality [23]. The 3D models of the GTs have been finally validated by generating a Ramachandran plot using PROCHECK (http://www.ebi.ac.uk/thornton-srv/software/PROCHECK/, accessed on 1 May 2021) that finds out the energetically allowed regions for backbone dihedral angles ψ against φ of the amino acids present in protein structures [24].

### 2.5. Functional Analyses of the Enzymes

To analyze the functional protein–protein interaction networks, web-based server, STRING v10.5 (https://string-db.org, accessed on 1 May 2021) was used [25]. These functional networks help to decipher complex molecular mechanisms of interactive pathways of the proteins of interest [26]. Every single interaction helps to understand the actual relation between interrelating domains present in our protein of interest with different un-annotated proteins of the biological pathways [25]. A computational tool, PHOBIUS (https://phobius.sbc.su.se/, accessed on 1 May 2021), was run to predict the combined transmembrane topology and signal peptide present in the chosen protein structures. Topology prediction helps to find the membrane-spanning domain of a protein, i.e., whether the enzyme is present in the cytosol or in the membrane. The integrated set of web-based tool MEME suite (Multiple Extraction-Maximization for Motif Elicitation) tool (http://meme-suite.org/, accessed on 1 May 2021) was used to search and characterize motifs present in the protein structures. Protein motifs apprehend the molecular interactions within a cell, which is an important biological function [27]. One more computational server PredictProtein (https://predictprotein.org/, accessed on 1 May 2021) [28] was applied to analyze the detailed structure and function of the chosen proteins of interest.

### 2.6. Selection and Optimization of Ligand with the Target Proteins

The ligand molecule uridine diphosphate-glucose (UDP-glucose) was converted to canonical SMILES using PubChem tool for molecular docking (https://pubchem.ncbi.nlm.nih.gov/compound/8629#section=InChI, accessed on 1 May 2021). Online server SwissADME (http://www.swissadme.ch/index.php, accessed on 1 May 2021) predicted the physicochemical properties, drug-likeness nature, pharmacokinetic, and medicinal properties of the ligand molecule [29]. Further, molsoft service (http://molsoft.com/mprop/, accessed on 1 May 2021) has confirmed the physicochemical properties of the ligand molecule regarding its molecular and drug-likeness nature. Later, Molinspiration cheminformatics toolkit (https://www.molinspiration.com/, accessed on 1 May 2021) was used to process the property (bioactivity and 3D structure) of the chosen ligand for docking [30]. This kit offers a broad range of cheminformatics tools supporting molecule manipulation and processing, including the normalization of molecules, molecule fragmentation, molecular modeling, and drug design. It directs the calculation of important molecular properties, such as logP, polar surface area, the number of hydrogen bond donors, and acceptors, and prediction of bioactivity score for the most important drug targets [30]. POCASA 1.1 (http://altair.sci.hokudai.ac.jp/g6/service/pocasa/, accessed on 1 May 2021) has been used to analyze the total number of 3D pockets present in all the three chosen protein sequences. Molecular editing of the ligand was done using ACD/ChemSketch from ACD/Labs (https://www.acdlabs.com/resources/freeware/chemsketch, accessed on 1 May 2021). The edited ligand structure was saved as MDL Molfiles. OpenBabelGUI software was used to convert the MDL Molfiles into PDB format for further molecular docking study (https://openbabel.org/docs/dev/GUI/GUI.html, accessed on 1 May 2021).

### 2.7. Molecular Docking to Ensemble Protein Structure

For the docking analysis, Swiss-PDBViewer 4.1.0 was run for the structural energy minimization of the ligand (https://spdbv.vital-it.ch/, accessed on 1 May 2021). Molecular docking helps to predict the interaction between target enzymes (protein) with small molecules (ligand). Further, AutoDock 4.0 was run to do the dock based on the Lamarckian genetic algorithm [31]. Using this e-server, polar hydrogens (AD4 type atoms) and Kollman charges were added to the protein structures. The center grid box was also prepared with proper orientation and grid spacing. The docking algorithm was finally run using Cygwin64 Terminal creating a .dlg file (https://www.cygwin.com/, accessed on 1 May 2021). Lastly, based on the binding energy, UCSF Chimera 1.8.1 software was employed for the visualization and analysis of the docking [21].

## 3. Results and Discussion

### 3.1. Accession of Target Proteins

Sequences of EPS producing glycosyltransferase (GT) enzymes from increasing growth temperature ranges (i.e., mesophile, thermophile, and hyperthermophile) have been retrieved from NCBI Protein. Depending on background survey related to significant earlier studies on EPS production and reports on the presence of GT operon in it, three specific bacterial strains [Mesophile: *Lactiplantibacillus plantarum* (QDX85283, also earlier known as *Lactobacillus plantarum*); Thermophile: *Rhodothermus marinus* (WP_012843084); Hyperthermophile: *Thermus aquaticus* (WP_003048358)] from each category have been chosen to further understand the in silico structural-functional relationship of the GTs.

### 3.2. Primary Analysis of Physicochemical Parameters and Structure

Computational study on physicochemical parameters of any protein helps to delineate the behavioral overview and nature of the proteins of interest. In this study, GT sequences from three different sources (based on increasing temperature for growth; mesophiles, thermophiles, and hyperthermophiles) were primarily characterized by basic physicochemical parameters such as the number of amino acids, molecular weight, theoretical pI, instability index, aliphatic index, extinction coefficient, and GRAVY (Table 1). Here in our study, pI value is high enough for both thermophile (9.85) and hyperthermophile (9.04) but low for mesophilic isolate (6.18). The high pI value indicates basic amino acid composition, whereas the low pI value indicates the acidic nature of the amino acids present in the protein [26]. Thus, our results indicate that GTs retrieved from thermophilic and hyperthermophilic bacteria are composed more of basic amino acids while GT isolated from mesophilic bacteria consisted more of acidic amino acids. In the case of the instability index, the value lower than 40 indicates the stable nature of the protein [32]. In this context, GT isolated from hyperthermophilic bacteria (40.53) was relatively unstable in comparison to the thermophilic one (31.28); but GT from the mesophilic source (16.34) was found to be quite stable in nature. The higher aliphatic index denoted the thermal stability of globular proteins based on amino acid compositions, i.e., the presence of more aliphatic amino acids like alanine (Ala), valine (Val), and leucine (Leu) groups. According to our study, enzymes isolated from the thermophile were more thermally stable due to the presence of more aliphatic amino acids than hyperthermophile and mesophile as stated previously by Haki and Rakshit [33]. Furthermore, it is interesting to observe that enzymes from all three different growth temperature ranges were hydrophilic in nature as the negative GRAVY value (−0.419, −0.210, and −0.223 respectively from mesophilic, thermophilic, and hyperthermophilic origin) denoted better interaction of the protein with water [34,35].

**Figure 1:**
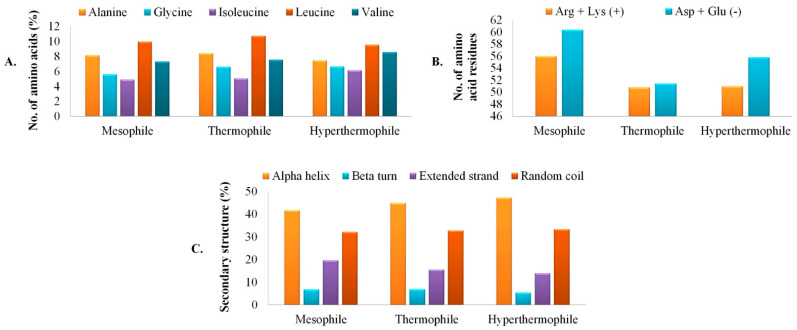
Graphical representation of primary, secondary, and tertiary structures of glycosyl transferase enzyme from three different classes: mesophile: *Lactiplantibacillus plantarum*; thermophile: *Rhodothermus marinus*; hyperthermophile: *Thermus aquaticus*, where (**A**). Percentage of five common amino acids contributing to structural properties, (**B**). Comparison in the composition of positively charged (Arg+Lys) and negatively charged (Asp+Glu) amino acid residues, (**C**). Comparison of secondary structures.

By analyzing the primary structure, it had been found that aliphatic amino acid Leu was commonly present in all the proteins in considerable numbers along with Ala and Val. Other than that, isoleucine (Ile) and glycine (Gly) were also commonly found in all the three different origin GTs. Interestingly, the presence of zero cysteine (Cys) residues in *L. plantarum* followed by thermophile *R. marinus* (1) and then hyperthermophile *T. aquaticus* (8), denotes ancient metal-binding property of the enzymes [36]. In the thermophile origin GT, a scarcity of strong polar residues were observed in comparison to the mesophile. This occurrence may be supported by the fact that in thermophile there is significant evolutionary pressure to offload destabilizing polar amino acids, to decrease the entropy cost of the side chain burial, and to eliminate thermally sensitive amino acids [37]. Mn^2+^ dependent activity of GT is already reported earlier [1]. Thus, from our study it may be delineated that the mesophilic GT may demonstrate a divalent metal ion-independent activity, which supports the wet-lab results of Thiyagarajan et al. [38]. Compositional differences in primary structure configuration and the top five contributing amino acids in the GTs are represented in Figure 1A. The total number of both positively and negatively charged amino acids was found to be highest in the case of mesophile compared to thermophile and hyperthermophile. Where as thermophile was found to be the only candidate balancing the number of both groups of charged amino acids (Figure 1B). Overall, GTs isolated from all the three different temperature ranges contained aliphatic amino acids with net negative charge.

### 3.3. Secondary Structure Assessment

The enzyme GT isolated from three different sources (based on increasing growth temperatures) were commonly accounted for α-helices (41.75%, 44.83%, and 47.34% respectively from the mesophilic, thermophilic, and hyperthermophilic GTs), followed by random coils (32.12%, 32.73%, and 33.25% respectively for mesophilic, thermophilic, and hyperthermophilic origin GTs) (Figure 1C). Higher percentages of amino acids taking part in α-helix and random coil formation indicate the protein with true enzymatic function along with structural flexibility and enzymatic turnover [39]. The relationship of protein stability with the increased presence of the classical repetitive secondary structure α-helices is already a well-known phenomenon. Unusually, stable helix formation is reported from short-Ala based proteins [40]. In our study, hyperthermophilic origin GT has the lowest Ala residues present compared to the other two sources. Non-native states of proteins are of increasing research interest because of their relevance to protein folding, translocation, and stability. The random coil is one such non-native state [41]. As observed from the complete secondary structure prediction, GT from the hyperthermophilic origin has less propensity to be denatured compared to the mesophiles and thermophiles, which indicates very high structural rigidity for the hyperthermophilic origin GT. The formation of extended strands in proteins is often associated with the formation of β-sheets. The presence of high proline (Pro) residues is a factor that commonly results in more extended strand formation [42], but in this case, GTs from all three sources did not have a significant presence of Pro residues. Steric interference between the main chain and side chains of a protein is known to be relieved in extended strand conformations, but hydrogen bonds are sacrificed in this state, decreasing the overall protein stability [43]. As observed from our results, the percentage of extended strands is lowest in the hyperthermophiles, followed by thermophiles and mesophiles, again confirming the high structural rigidity of the *T. aquaticus* origin GT. β-turns play an important role in mediating interactions between enzymes and their ligands/receptors, thus playing a great role in the functional activity of a protein [44]. In our study, it has been interestingly observed that though hyperthermophilic GT was highly stable and rigid in its conformation, mesophilic and thermophilic origin GTs demonstrated better plasticity. Nearly 6.8% β-turns are present in mesophilic and thermophilic origin GTs, while the hyperthermophile has considerably low (~5.4%.) This specifies a more enzyme-ligand interface and better functional plasticity of the GTs from moderate temperature sources.

After analyzing the total number of hydrogen bonds present in each of the retrieved GT sequences, it was found that the presence of hydrogen bonds was highest in the case of mesophiles (355), while it was nearly similar for both hyperthermophiles (292) and thermophiles (290). Hydrogen bonds are reported to provide energy (5 Kcal mol^−1^) in maintaining the configuration stability of the proteins. Later it has also been found that hydrogen bonds in α-helices provide more energy (8 Kcal mol^−1^) to the protein structure [45]. Hydrogen bonds between the amino acids favor protein folding, while conformational freedom of the protein is directed upon unfolding [37]. However, in our study, mesophilic origin GT was observed with the maximum presence of hydrogen bonds. The most striking difference among them is the increased hydrophobicity of thermophilic transmembrane helices, possibly reflecting more stringent hydrophobicity requirements at high temperatures [37], which is already demonstrated in the secondary structure results with the maximum rigidity in the case of hyperthermophiles. Therefore, it can be said that the contribution of hydrogen bonds in structural stability is contextual. In this study, more α-helix along with hydrogen bonds make the proteins structurally stable in nature.

### 3.4. Assessment of the Three Dimensional Structure, Modeling, and Validation

**Figure 2:**
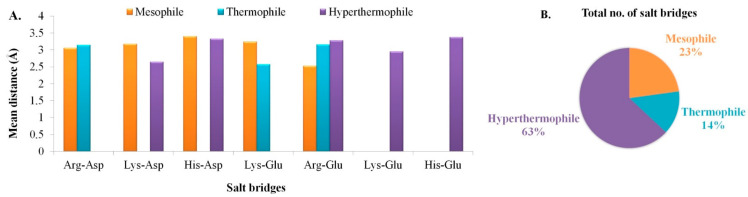
Salt bridge analyses, where: (**A**) Salt bridge composition along with mean distances, and (**B**) Comparison in the number of salt bridges present in three different classes.

The prediction of protein three-dimensional structure from amino acid sequence has been a big challenge in computational biophysics [46]. The reason for which there has been growing interest in protein biotechnology in terms of in silico three-dimensional structure prediction, modeling, and structural validation. Salt bridges play an important role in the prediction of protein’s three-dimensional structure by being involved in oligomerization, molecular recognition, allosteric regulation, domain motions, flexibility, thermostability, and more [47,48]. Salt bridges are recognized if at least one Asp or Glu side-chain carboxyl oxygen atom and one side-chain nitrogen atom of Arg, Lys, or His are within a distance of 4.0 Å. Salt bridge is a type of non-covalent ionic bond that plays an important role in the stabilization of protein structures [47]. The numbers of salt bridges existing in the tertiary structure of our target GTs and the mean interatomic distance of that salt bridges formed between the amino acid residues was computed using the ESBRI interface and are depicted in Figure 2A,B. Our study revealed that the interatomic distances of all the salt bridges were less than 7 Å and proved to be stable in nature. GTs isolated from hyperthermophiles had shown more salt bridges in comparison to the mesophiles and thermophiles, which may be supported by the fact that due to unfavorably high temperature, proteins have formed more ionic interactions to make it structurally stable. Arg-Glu, followed by Arg-Asp and Lys-Glu were the dominating salt bridges observed in all the three sequences. Arg-Glu > Arg-Asp > Lys-Glu; this is the tendency of salt bridge pairs favorability on ∝-helix stabilization and slowing down destabilization of helix structures [49,50], just as observed in the case of our target hyperthermophile-origin GT.

**Figure 3:**
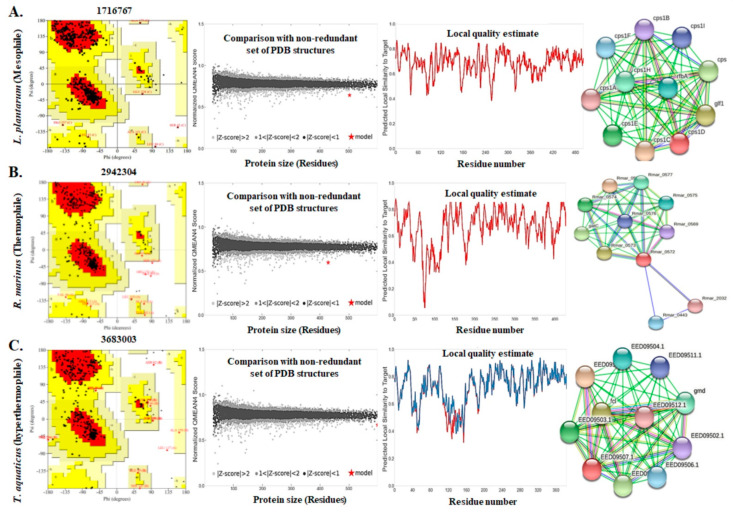
Quality appraisal from Ramachandran plots, Evaluation of Z-score, local model quality estimation, and functional assessment by protein–protein interaction networks of glycosyl transferase enzymes isolated from three different classes: (**A**) mesophile: *Lactiplantibacillus plantarum*; (**B**) thermophile: *Rhodothermus marinus*, and (**C**) hyperthermophile: *Thermus aquaticus*.

The protein structures were validated again using the ERRAT server that compares statistics of non-bonded interactions between different atoms of highly refined crystallographic protein structures. It plots their error function value against the position of 9-residue sliding window [22]. The overall quality factors of GTs isolated from three different categories (84.48 for mesophile; 80.77 for thermophile; 85.05 for hyperthermophile) were found to be satisfactory. This computational analysis also confirms the crystallographic 3D structures of the proteins. Furthermore, the SAVES server had shown the excellence of the protein structures by providing 3D-1D score based on the structural ratio. Based on these scores (83.70 for mesophile; 92.54 for thermophile; 87.53 for hyperthermophile), all the query proteins were confirmed to have stable crystallographic structure; while the GT isolated from thermophile had a more stable crystallographic structure than the other two categories (Table 1). For homology modeling and global quality assessment of the target proteins, the SWISS-MODEL QMEAN tool was used. The structure of GTs were compared with non-redundant PDB structures in order to predict the protein quality model. The QMEAN score value within 0–1 [51] and protein Z-score value < 1 [52] confirm a high-quality model. According to the Z-scores predicted in our study, GTs isolated from thermophile (−4.98) had higher model quality in comparison to mesophile (−4.01) and hyperthermophile (−2.59), respectively. Target template alignment was predicted by local model quality estimation and structural interpretation using the SWISS-MODEL as demonstrated in Figure 3. The graph showed local model similarity to the target protein by comparing it with non-redundant PDB structures. The three-dimensional structure of GT was justified stereochemically by PROCHECK [24]. It produced Ramachandran plots by arranging amino acid residues against φ-ψ torsion angles. The first quadrant of the Ramachandran plot contains the allowed region having rare left-handed α-helices. The second quadrant stereochemically allows the most favorable β-strand conformations. The third quadrant of the Ramachandran plot holds right-handed α-helices; whereas the most unfavorable conformation or disallowed region is the fourth quadrant [24]. According to our study, Ramachandran plot of GTs isolated from their different bacterial sources had shown that amino acids residues (89.7% for mesophile; 83.4% for thermophile, and 86.8% for hyperthermophile) were mostly in favorable region while very few or no residues in disallowed region, making it definite that the protein had satisfactory model quality (Figure 3A–C).

### 3.5. Functional Analyses of the Enzymes

In silico functional analysis of the proteins are used to assign biological or biochemical roles to the query proteins. In the present study, protein–protein interaction networks, protein topology, functional domains and motifs present in the target GT sequences have been studied as a part of functional analyses. Protein–protein interactions were observed using the STRING database (Figure 3). GTs from all three different bacterial sources had shown considerable first shell interaction with other proteins. The colored nodes represent each 3D-structure of proteins, which may be generated by post-transcriptional modification of the same protein-coding gene locus. This association jointly contributes to any shared function without binding physically with each other [53]. In our study, GT produced by mesophilic *L. plantarum* has shown more experimentally determined known interactions followed by *T. aquaticus* and *R. marinus* produced GTs. In addition, a varied number of gene neighborhood interactions are predicted in all the three GTs of interest, indicating the different conserved topological structure of all the three proteins of interest from different sources; while the predicted functional partners of all these three enzymes commonly consist of different EPS/CPS biosynthesis-related genes in different sources. Particularly in the case of mesophile, from STRING analyses, it can be easily depicted that 10 nodes were connected with 55 different edges (node score range 0.899–0.758), whereas the expected number of edges was 10 with an average node degree value of 10 (i.e., each node had at least 10 interacting nodes). Average local clustering coefficient was predicted to be 1 with a protein–protein interaction (PPI) enrichment *p*-value <1.0e−16. This result means that proteins of interest have more interactions among themselves than expected for a random set of proteins of similar size. Such type of interaction enrichment indicates that the protein of interest has a partial biological connection. Glucose-1-phosphate thymidylyltransferase (rfbA, node score 0.820) is only predicted annotated interacting protein which catalyses formation of dTDP-glucose from dTTP and glucose 1-phosphate, in addition with pyrophosphorolysis as well. In the case of thermophile, 11 nodes were observed to be connected with 23 different edges (node score range 0.934–0.566), whereas the expected number of edges was 11 with an average node degree value of 4.18 (i.e., each node had at least 4.18 interacting nodes). The average local clustering coefficient was 0.872 with a PPI enrichment *p*-value 0.000989. This result also indicates similar interactions, such as mesophile, against a random set of proteins. Closest annotated interacting protein murG with score 0.934 functions as undecaprenyl-PP-MurNAc-pentapeptide-UDPGlcNAc GlcNAc transferase to form the cell wall. Another two interacting proteins murD (0.605) and murA (0.566), are involved in polysaccharide formation. Finally, in the case of hyperthermophile, 10 nodes were connected with 55 different edges (node score range 0.900–0.684), whereas the expected number of edges was to be 10 with an average node degree value of 10, i.e., each node had at least 10 interacting nodes. The average local clustering coefficient was predicted to be 1 with a PPI enrichment *p*-value <1.0e-16. The interaction against a random set of proteins was similar to that of mesophile and thermophile. Closest annotated interacting protein GDP-L-fucose synthase (fcl) had the shortest node score 0.900 that functions in catalyzing two-step NADP-dependent conversion of GDP- 4-dehydro-6-deoxy-D-mannose to GDP-fucose. Gmd (score 0.865) acts in catalyzing the conversion of GDP-D-mannose to GDP-4- dehydro-6-deoxy-D-mannose, EED09503.1 (score 0.865) is related to teichoic acid export, and EED09502.1 (score 0.761) is involved in dimerization of UDP-glucose/GDP-mannose by dehydrogenase function. Thus, from the overall PPI network study, it can be clearly predicted that GTs are a definite part of polysaccharide biosynthesis.

Another functional analysis-related web interface, PHOBIUS, helps in the prediction of signal peptides and transmembrane topology of amino acid sequences in a protein by reducing the cross-prediction errors. From the resultant probability plot of our study set, it was observed that the enzyme GT is present in a transmembrane form in all three classes of bacteria according to growth temperature. GT-related polysaccharide biosynthesis (EPS/CPS) is reported to have a transmembrane domain attached to the cell membrane involved in glycosyl transfer, sugar polymerization, and more [10,54]. Likewise, the MEME suite helps to find biologically functional motifs in the target protein sequences along with finding binding sites of the protein with other regulatory elements. Therefore, this study is reported to be important to locate the binding sites of ligand molecules with the target protein for increased functionality [55]. Motifs are basically signature sequences that aid in the identification of any protein. The e-value shows accuracy of the predicted motif; less the e-value, more the precision of the possible motifs [56]. From our study, it has been found that the GTs retrieved from all the three different environments based on growth temperatures (i.e., mesophile, thermophile, and hyperthermophile), e-value were less than 3.0e + 000. This indicates that the predicted motifs are accurate and biologically active. According to Bailey [55], MEME reports the occurrences of sites, consensus sequence, and the level of conservation at each position in the pattern. As it has been observed in our results, predicted motifs from all the three mesophile, thermophile, and hyperthermophile-origin GTs have demonstrated highly variable patterns in most of the positions. E-server PredictProtein analyses the proteins both structurally and functionally based on evolutionary information obtained from the PSI-BLAST search [57]. One of the PredictProtein tools determines solvent accessibility (RePROF) or accessible surface area (ASA) depending on the 3D-1D sequences, which is one of the predominant steps towards predicting the functionality of that protein [58]. Prediction of the ASA can be of two types; buried or exposed. While a 5% threshold may predict that a residue will be revealed, a 25% threshold may predict that the same residue will be buried. [59]. As it may be observed in the case of the three GTs from different sources, the ratio of buried and exposed residues are nearly similar, indicating stable protein–protein interactions and bioactivity. This also corresponds to the presence of a good amount of random coils, turns, β-structures, and helices in the enzymes [60]. Interestingly, the number of buried residues significantly increased from mesophile to thermophile to hyperthermophile. The buried residues usually form hydrophobic cores to maintain the structural integrity of proteins, while the exposed residues are highly related to protein functions. The result again confirms increasing structural rigidity in the following order mesophile > thermophile > hyperthermophile, while just the reverse order in case of functional plasticity. Another PredictProtein tool ConSeq identifies structurally and functionally important evolutionary conserved amino acids of a protein that are involved in binding of ligand and macromolecules, and protein–protein interactions by means of predicting the conservation score [57]. As it has been found in all three of our chosen GTs from different growth temperatures, these are often evolutionarily conserved and are more likely to be accessible to the solvent, while their core residues probably are pivotal in protein folding [61]. Interestingly more “low conservation score (1–3)” has been observed for mesophile, while “intermediate conservation score (4–6)” for thermophile followed by “high conservation score (7–9)” for the hyperthermophile. The results correlate to RePROF analysis of better surface accessibility for the mesophile and most maintained core for the hyperthermophile, while the thermophile creates a perfect balance between both. The final PredictProtein tool PROFbval predicts residue mobility through a relative B-value that is significant for improved functionality of a protein-based on its amino acid sequences [57]. The relative B-value was commonly found to be “intermediate (31–70)” for all the three target GT sequences. This indicates the presence of moderately flexible residues in the protein surface of all the three GTs from varied growth temperatures, a key to maintain its basic function in any environment [62].

### 3.6. Selection and Optimization of Ligand with the Target Proteins

**Figure 4:**
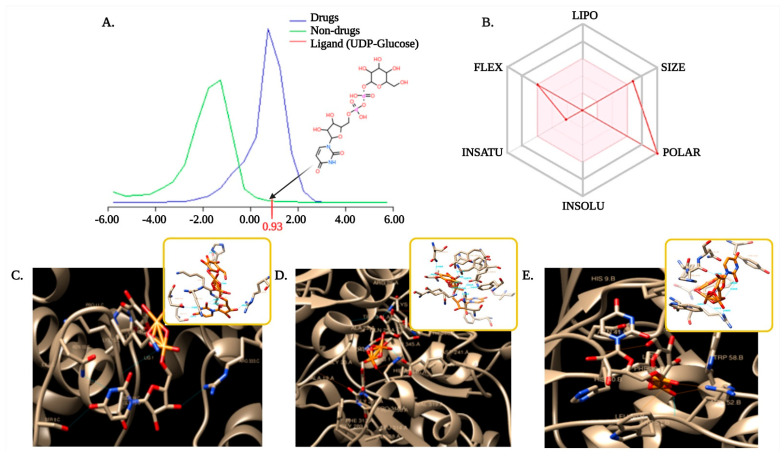
Ligand preparation and molecular docking, where: (**A**) chemical structure, drug-likeness score (0.93); (**B**) ESOL model representing properties of the UDP-glucose ligand as a polar molecule, UDP-glucose-glycosyl transferase interactions in three different cases: (**C**) mesophile: *Lactiplantibacillus plantarum*, (**D**) thermophile: *Rhodothermus marinus*, and (**E**). hyperthermophile: *Thermus aquaticus*.

Substrate binding affinity is a complex characteristic determined by balancing various molecular and structural properties to determine whether a specific molecule is similar to any known substrates or enzymes. In chemoinformatics, four primary parameters namely, absorption, distribution, metabolism, and excretion (ADME), are required for ligand target binding assessment [29,63]. As enzymes play a pivotal role in all biological systems and often have high industrial value, like that of GTs in bacterial polysaccharides production, it is crucial to predict the correct, mechanistically appropriate binding modes for substrate and product [64]. The drug-likeness model score of the UDP-Glucose ligand was found to be 0.93, as might be observed in Figure 4A. Drug likeliness is a complex qualitative factor that determined bioavailability of a ligand for its substrate [65]. A positive scoring indicates our ligand fit for the substrate interaction. According to the ESOL model, the selected ligand UDP-Glucose (C_15_H_24_N_2_O_17_P_2_) of molecular weight 566.30 g/mol has 17 hydrogen bond acceptors and 9 hydrogen bond donors. This ligand molecule is also highly soluble in water, suggesting the ligands as very polar in nature (Figure 4B). The ligand screening was done through Bayesian statistics using the Molinspiration toolkit that helped to find molecule bioactivity score. It is known that molecules with bioactivity scores between −3 to 3 are active, while the score less than −3 is inactive. Molecules with high activity scores are much probable to be highly active in nature [66]. In our present study, the bioactivity score of important drug classes like GPCR ligand was 1.07, while score for the enzyme inhibitor was 1.32, which means the selected ligand molecule was intermediately bioactive in nature with the chosen GTs. Bacterial GTs are already established with the idea to utilize Uridine diphosphate (UDP)-glucose for polysaccharide (EPS/CPS/LPS) biosynthesis [67,68]. Hence, for complete functional elucidation of temperature-dependent expression of bacterial GTs, it is significant to observe its ligand (UDP-glucose) binding affinity. Selection and preparation of the ligand for enzyme binding is a preliminary step to move forward to docking analysis.

### 3.7. Molecular Docking to Ensemble Protein Structure

Molecular docking effectively helps to predict the relationship of protein structure with ligand molecules [69]. The biological activity of small molecules against enzymes can be determined from docking calculation [70]. Docking is a theoretical simulation method based on computational techniques, has a promising role in medical chemistry like drug designing [71] or for screening in food science. Here, in our study, semi-flexible molecular docking has been performed by using a fixed small molecule and GTs from three different sources. Acylation of L-lysine at a molecular level via semi-flexible docking simulation help to calculate the interactions [71]. AutoDock tool analyzes the biomolecular complexes using computational docking, where the first step is to prepare the coordinate files for the docking molecule and the target molecule. It is followed by the calculation of the affinity grid for the target molecule. In the final step, the docking molecule is docked with the affinity grid to analyze the result [72]. As water molecules compete with the ligands to form hydrogen bonding, therefore, removing the unfavorable molecules improves the binding [73]. In this aspect, AD4 uses polar hydrogens for docking analysis. Compared to the traditional genetic algorithm for docking, the Lamarckian genetic algorithm can handle ligands with more degrees of freedom and is the most efficient, reliable, and successful method [31]. Binding energy is an important factor for successful docking with active ligand mixed to inactive decoys [74]. According to our results, it was observed that ligand UDP-glucose (chosen and optimized) were respectively binding to GTs with binding energy between −5.85 to −7.72 for mesophiles; −5.57 to −10.70 for thermophiles; −7.85 to −9.90 for hyperthermophiles. Based on a forced-field-based approach, ligand molecules with the highest binding affinities were most favorable for molecular docking [31]. Our results also demonstrated that the number of hydrogen bonds involved to make a stable interaction between ligand and enzyme is significantly different for different enzymes. It was high for thermophile (10), followed by mesophile (7) and hyperthermophile (6), respectively; which indicates stable and stronger enzyme–substrate interaction in thermophile *R. marinus* origin GT compared to mesophile and least for hyperthermophile (Figure 4C–E). An increase in the number of hydrogen bonds is already reported earlier to strengthen the interaction as stronger and more stable [73]. Distance geometry is another important parameter for analyzing the biological activity of ligand molecules [75]. The average distance of these interactive H-bonds varies between 2.407 Å–3.471 Å in our study set. Asp, Lys, His, and Arg are most commonly found to be involved in the formation of H-bonds for ligand-macromolecule interaction. Interestingly, in the case of thermophile *R. marinus* origin GT, other than the mentioned amino acids, Phe, Ala, Glu, Tyr, and Ser were also involved in the formation of H-bonds. From the overall findings of molecular docking analysis, ligand affinity, and stability were found to be higher in the case of thermophile followed by mesophile and hyperthermophile. Docking is an integrated part of computational biology that considers the bioactivity and toxicity of small molecules. Thus, molecular modeling is an approach in assessing the drug-likeness property, which will help to use that small molecule in food/pharmaceutical industry [76]. This study illustrates structural approaches to assess the interspecific differences in a functional perspective. Though there have been several earlier reports on the involvement of GT enzyme in polysaccharide biosynthesis for hyperthermophiles, thermophiles, or mesophiles; but our study has been the first novel approach to understand the temperature-dependent structure-function relationship of bacterial polysaccharide synthesizing GTs. As mentioned earlier, to the best of our knowledge, this is the first study on EPS biopolymer producing GT enzymes from a broad range of temperature-adapted bacterial strains. From the overall analysis, it may well be summarized that while the structural integrity of GT increases significantly from mesophile to thermophile to hyperthermophile, the structural plasticity runs in an opposite direction from hyperthermophile to thermophile to mesophile. The interesting temperature-dependent structural property has directed the GT-substrate (UDP-glucose) interactions in a way that thermophile has demonstrated better binding affinity with an increased number of hydrogen bonds and stabilizing amino acids, suitable for any future industrial usage in EPS production.

## 4. Conclusions

Bacteria often express precise and delicate adaptations in different environments. While some like it hot, few like it hotter, and the others like it normal. Molecular adaptation occurs even with high sequence and structural similarities among the enzymes from highly varied temperature sources [77]. Electrostatics play an important role for survival in high temperatures by structural adaptations of the proteins. For example, a higher number of salt bridges in hyperthermophile in comparison to thermophile and mesophile probably resist the active site disorder due to heat shock. In addition, despite heat adaptation and structural rigidity, possibly hyperthermophiles counter its active site with significantly less deviation in sequences [77]. From our present study, it can be concluded that the structural integrity of GT enzyme increased significantly from mesophile > thermophile > hyperthermophile, while the structural plasticity runs in an opposite direction from hyperthermophile < thermophile < mesophile. The interesting temperature-dependent structural and functional property has directed the glycosyl transferase–UDP-glucose interactions in a way that thermophile origin GT demonstrated better binding affinity with an increased number of hydrogen bonds and stabilizing amino acids. GTs are a wide class of enzymes that transfers sugar moiety, playing a key role in bacterial EPS biosynthesis. In recent years, increased demand for bacterial EPSs is observed in different industries like foods and pharmaceuticals. As the industrial application of the EPSs largely depends upon its thermal stability and structural plasticity in high temperatures, the results from this study may direct utilization of thermophile-origin GT as a suitable candidate for industrial production of bacterial EPS in the future.

## Figures and Tables

**Table 1:** Comparative physicochemical characteristics and quality assessment of the three chosen bacterial glycosyl transferase enzyme from mesophile, thermophile, and hyperthermophile.

Serial No.	Bacterial Isolates	Physicochemical Characters						Quality Assessment Scores			
		No. of AA	Theoretical PI	MW (KDa)	II	AI	GRAVY	3D-1D Score (%)	ERRAT Quality Factor	QMEAN Z- Score	AA in FR of Ramamchandran Plot (%)
1	*Lactobacillus plantarum*	514	6.18	58.346	26.34	77.88	−0.419	83.70	84.4898	−4.01	89.7
2	*Rhodothermus marinus*	443	9.85	50.116	31.28	94.09	−0.21	92.54	80.7786	−4.98	83.4
3	*Thermus aquaticus*	396	9.04	44.256	40.53	88.21	−0.223	87.53	85.0575	−2.59	86.8

Where AA, amino acids; MW, molecular weight; II, instability index; AI, aliphatic index; GRAVY, grand average hydropathy; FR, favorable region.

## Data Availability

The data presented in this study are available on request from the corresponding author.
